# Dietary wheat and reduced methane yield are linked to rumen microbiome changes in dairy cows

**DOI:** 10.1371/journal.pone.0268157

**Published:** 2022-05-19

**Authors:** Keith W. Savin, Peter J. Moate, S. R. O. Williams, Carolyn Bath, Joanne Hemsworth, Jianghui Wang, Doris Ram, Jody Zawadzki, Simone Rochfort, Benjamin G. Cocks

**Affiliations:** 1 AgriBio Centre, Agriculture Victoria Research, Bundoora, Victoria, Australia; 2 Agriculture Victoria Research, Ellinbank, Victoria, Australia; United States Department of Agriculture, Agricultural Research Service, UNITED STATES

## Abstract

Fermentation of pasture grasses and grains in the rumen of dairy cows and other ruminants produces methane as a by-product, wasting energy and contributing to the atmospheric load of greenhouse gasses. Many feeding trials in farmed ruminants have tested the impact of dietary components on feed efficiency, productivity and methane yield (MeY). Such diets remodel the rumen microbiome, altering bacterial, archaeal, fungal and protozoan populations, with an altered fermentation outcome. In dairy cows, some dietary grains can reduce enteric methane production. This is especially true of wheat, in comparison to corn or barley. Using a feeding trial of cows fed rolled wheat, corn or barley grain, in combination with hay and canola, we identified wheat-associated changes in the ruminal microbiome. Ruminal methane production, pH and VFA concentration data together with 16S rRNA gene amplicon sequences were used to compare ruminal bacterial and archaeal populations across diets. Differential abundance analysis of clustered sequences (OTU) identified members of the bacterial families *Lachnospiraceae*, *Acidaminococcaceae*, *Eubacteriaceae*, *Prevotellaceae*, *Selenomonadaceae*, *Anaerovoracaceae* and *Fibrobacteraceae* having a strong preference for growth in wheat-fed cows. Within the methanogenic archaea, (at >99% 16S rRNA sequence identity) the growth of *Methanobrevibacter millerae* was favoured by the non-wheat diets, while *Methanobrevibacter olleyae* was unaffected. From the wheat-preferring bacteria, correlation analysis found OTU strongly linked to reduced MeY, reduced pH and raised propionic acid levels. OTU from the genera *Shuttleworthia* and *Prevotella_7* and especially *Selenomonadaceae* had high anti-methane correlations. An OTU likely representing (100% sequence identity) the fumarate-reducing, hydrogen-utilising, rumen bacterium *Mitsuokella jalaludinii*, had an especially high negative correlation coefficient (-0.83) versus MeY and moderate correlation (-0.6) with rumen pH, strongly suggesting much of the MeY suppression is due to reduced hydrogen availablity. Other OTU, representing as yet unknown species from the *Selenomonadaceae* family and the genera *Prevotella_7*, *Fibrobacter* and *Syntrophococcus* also had high to moderate negative MeY correlations, but low correlation with pH. These latter likely represent bacterial species able to reduce MeY without causing greater ruminal acidity, making them excellent candidates, provided they can be isolated, for development as anti-methane probiotics.

## Introduction

While farmed ruminants such as cattle can feed on various forage species and grains, they do so at a cost to the environment. A major concern for global warming is the greenhouse gas methane emitted by ruminants such as sheep, goats and cattle [1, 2]. This gas is a natural product of the fermentation process occurring in the rumen of animals digesting feeds such as grasses and grains.

Many dietary interventions have been tested in farmed ruminants, such as dairy cows, aiming to reduce methane emissions or improve feed efficiency. Different pasture and forage types have been evaluated along with a variety of edible materials such as various grains [3], plant extracts and chemical additives such as 3-nitrooxypropanol [4–6]. The benefits and drawbacks of a variety of dietary additives including wheat and *Asparagopsis* seaweed have been recently reviewed [7].

An important theme seen in many of these studies has been the redirection of metabolic hydrogen away from production of methane and more toward propionate [8] or other reduction reactions. The type of grain fed may influence methane production from the rumen, but it remains unclear what impact diets have on this microbiome or how the effect may be caused. Is it due to starch degradation rate for example, or some unidentified factor in one grain or another? Perhaps also the outcome of feeding different diets may depend on the pre-existing state of the ruminal microbial population [9]. Different forages and grains have been compared and exotic additives tested in an effort to manipulate the fermentation process occurring in the rumen [6]. Several feeding experiments have included an investigation of the impact of dietary changes on the rumen microbiome [10]. Comparisons have been made using the commonly available grains such as corn, wheat, oats and barley, resulting in some promising, but sometimes contradictory, reductions in methane production. For example, in sheep, a diet containing wheat was found to generate more methane than a diet including corn [11], whereas in dairy cows the opposite is true. Moate et al. [12] found that inclusion of wheat in the diet reduced methane production significantly when compared to dietary inclusion of corn or barley. Here we used 16S rRNA sequencing to study the rumen microbiome of dairy cows fed, over a 3-week period, with either barley, corn or wheat. Understanding the rumen microbiome is key to understanding rumen fermentation and the changes it undergoes in response to diet. We hypothesise that dairy cows fed on diets supplemented with different grains would have different rumen microbiome populations and that such microbial differences would explain the lower methane production seen with the diet supplemented with wheat compared to barley or corn.

## Materials and methods

### Ethics approval and consent to participate

All cows were maintained in the research herd at the Agriculture Victoria Research Ellinbank Centre, 1301 Hazeldean Road, Ellinbank, Victoria, Australia. All experiments were conducted in accordance with the Australian Code of Practice for the Care and Use of Animals for Scientific Purposes (National Health and Medical Research Council 2013). Approval to proceed was obtained from the DJPR Agricultural Research and Extension Animal Ethics Committee. No anaesthesia, euthanasia, or any kind of animal sacrifice was part of the study.

### Diet

The rumen samples collected in this study are from the experiment described previously on ruminal methane [12]. Cows were fed on hay, canola meal and grains as described [12]. Essentially the cows were fed 55% hay + canola meal + minerals and supplemented with 45% rolled grain (either corn, barley or wheat). The timeline of the feeding trial is summarised in S1A Fig. Rumen samples for microbiome analysis were collected in parallel to those used for chemical analysis and stored at -80C until processing for DNA sequencing.

Thirty-two lactating, multiparous Holstein-Friesian cows, including 12 fistulated cows, were assigned to one of 4 blocks, each block consisting of 5 non-fistulated and 3 fistulated cows. Each block was balanced for cow age, weight and days in milk (DIM) according to Baird [13] then randomly assigned to one of four dietary treatments:

1. CRN, comprising ~9.0 kg DM of chopped alfalfa hay, ~9.0 kg DM of single-rolled corn, 1.8 kg DM cold pressed canola meal, and 0.2 kg DM minerals fed as a total mixed ration (TMR) after milking
2. WHT, comprising ~9.0 kg DM of chopped alfalfa hay, ~9.0 kg DM of single-rolled wheat, 1.8 kg DM cold pressed canola meal, and 0.2 kg DM minerals fed as a TMR after milking.
3. SRB, comprising ~9.0 kg DM of chopped alfalfa hay, ~9.0 kg DM of single-rolled barley (barley_1), 1.8 kg DM cold pressed canola meal, and 0.2 kg DM minerals fed as a TMR after milking.
4. DRB, comprising ~9.0 kg DM of chopped alfalfa hay, ~9.0 kg DM of double-rolled barley (barley_2), 1.8 kg DM cold pressed canola meal, and 0.2 kg DM minerals, fed as a TMR after milking.

Each day at 0700hr and 1600hr, each of the 32 cows was fed in its individual feed stall provided with its specified feed and a supply of water.

Cold-pressed canola meal was included in these diets to ensure that the diets met the cows dietary requirements for protein and that the diet also supplied an amount of fat (i.e. > 500g fat/cow/day) equivalent to at least 50% of the expected milk fat yield of the cows.

The first week of the experiment constituted a covariate period during which all cows were fed a common diet comprising 6 kg DM/cow/day of rolled wheat and 16 kg DM/cow/day of alfalfa hay. The second week of the experiment constituted a transitioning period during which cows were transitioned onto their experimental diets. The third week of the experiment was an adjustment period during which the cows were fed their full experimental diets. The fourth and fifth (last) week of the experiment was an intensive period of measurements. Samples of ruminal contents were collected for microbiome analysis on the last day of week 5. Methane measurements were taken over the last 6 days of the trial.

Note that cows cn2319, wt9534 and wt9543 were treated with antibiotics (CepravinLC, Yodimaspen and Mastalone respectively) for mastitis in the month leading up to the transition period, before beginning the experimental diet treatments.

### Methane, pH and VFA measurements

Methane emissions were estimated using the SF_6_ tracer technique as described [14]. Using this method, methane measurement requires the controlled release of a tracer gas, sulphur hexafluoride (SF6) into the rumen. Gelatin-coated capsules containing about 2.8g SF6 were inserted into the rumen on day 24 of the trial. An animal’s methane emission was calculated from the measured gas-mixing ratio in eructated gases (SF6 tracer + CH4). Details can be found elsewhere [14]. Gas was collected using an evacuated canister connected to a suction tube near the cow’s mouth and was sampled for 24 hours every day over the last 6 days of the trial. The average of the methane production and consumed feed (dry matter intake, DMI) measurements over the last 6 days of the experiment was used to calculate the average gm CH4 per kg DMI (MeY) for this analysis. See S1A Fig which shows a timeline of the diet treatment and methane measurements. The pH of ruminal samples was measured immediately after collection using a pH meter (Mettler-Toledo. Also measured were ammonia using the QuikChem method 12-107-06-2-A from Lachat Instruments, D-lactate and L-lactate determined by measurement of NADH produced by -L or -D lactate dehydrogenase [15] and acetic, propionic, iso-butyric, butyric, iso-valeric, valeric, hexanoic and heptanoic acids determined using capillary gas chromatography as described by Packer et al. [16].

### Collection of ruminal content samples, genomic DNA extraction and 16S rRNA gene V4 amplicon sequencing

On day 35, the final day of the trial (21 days after the transition period), ruminal material was collected at approximately 11.30 AM from all cows using an oesophageal probe as described [4]. Rumen collection was by mouth using a suction tube. Two samples were collected, one frozen for bacterial microbiome analysis, one for chemical analysis. Samples (20ml) were kept frozen at -70°C until analysed. Genomic DNA was extracted from a 200 μL aliquot from each sample using the PSP Spin Stool DNA Kit (STRATEC Molecular GmbH, D-13125 Berlin, Germany) according to the manufacturer’s instructions.

Variable region 4 (V4) from the prokaryote 16S rRNA gene was amplified from the ruminal genomic DNA by polymerase chain reaction (PCR) using the F515/R806 primers (5’-GTGCCAGCMGCCGCGGTAA-3’/ 5’ -GGACTACHVGGGTWTCTAAT-3’) and associated published method [17], using Phusion DNA polymerase (ThermoFisher Scientific). The Illumina MiSeq system was used to generate DNA sequence data from the PCR fragments (251x 2 cycles, paired-end sequencing), according to the manufacturer’s instructions. The MiSeq fastq files generated are available at Figshare (Digital Object Identifier = 10.6084/m9.figshare.16689487) including those from no-DNA negative control PCR reactions.

The no-DNA controls were samples with the same detergent, buffer, DNA polymerase enzyme and other required ingredients, but without ruminal DNA. Instead, the DNA was replaced with ultra-pure water. They were processed in parallel with the DNA from ruminal samples. The ruminal DNA samples and 2 no-DNA controls were all subjected to the same PCR reactions and DNA sequencing together as a batch.

### Processing of 16S V4 amplicon DNA sequences

DNA sequence fastq files from MiSeq paired end (PE) sequencing were quality filtered using Trimmomatic software [18] using a sliding window method. Moving from one end of a sequence the 4-base window would cut the sequence if the average phred score of the 4 bases in the window was less than 15. If the remaining sequence was then less than 200 bases it was rejected. Quality score by base position plots of raw and filtered sequence data were generated by fastQC [19] and examined manually. Processing of fastq files, containing overlapping 5’ or 3’ end sequences, was carried out using basic unix utilities and the PANDAseq program [20] for assembly of paired-end sequences. Assembled sequence pairs were retained if they contained the 5’ and 3’ PCR primer sequences at their ends (using regular expressions based on the above sequences). Primer sequences were then removed from the sequence ends and sequences rejected if an ambiguous base (an N character) was present anywhere in the sequence. Unix utilities such as grep, gawk, sort and uniq were then used to aggregate and count replicate (100% identical) sequences, creating files of unique amplicon sequences of known abundance in fasta format.

All amplicon sequences found in the No-DNA controls were assumed to be contaminants and were removed from the ruminal sample sequences before any further analysis.

### Rarefaction analysis of microbiome sequences

Groups of merged paired-end sequences were randomly selected using the unix utility shuf, in increasing multiples of 2000. The groups were clustered using Uparse [21] at 97% identity and the count of OTU (clusters) plotted (using R) versus the total sequences in each group.

### Diversity and richness of microbiome sequences

Merged paired-end sequences were analysed for Shannon diversity index and Chao1 species richness using the R libraries vegan and fossil respectively. The values obtained were plotted for each ruminal sample.

### Taxonomy

Taxon assignments were carried out by aligning 16S rRNA V4 sequences to databases of 16S rRNA genes using Megablast [22]. Sequences were assigned to the family or genus level using the SILVA_138.1 database (https://www.arb-silva.de/documentation/release-138/ and https://www.arb-silva.de/no_cache/download/archive/release_138.1/Exports/). The file SILVA_138.1_SSURef_NR99_tax_silva.fasta.gz was converted into a blastable database using the makeblastdb software from NCBI. Sequences were assigned to the genus or species level (where possible based on sequence identity) using the Genbank 16S_ribosomal_RNA database (July 2020 https://ftp.ncbi.nlm.nih.gov/blast/db/).

### OTU clustering

Filtered and merged 16S rRNA V4 paired-end sequences that had then been aggregated (at 100% identity) to create unique sequences, were further clustered to Operational Taxonomic Units (OTU) using the Swarm2 software [23] using parameters -f (fastidious) -d 1 (default). The abundance of each OTU was calculated using the abundance of each unique sequence and the total sequence count within each cluster (each cluster is known as a swarm). A swarm resembles a phylogenetic tree where each layer or branch of the tree differs from the one above by a single nucleotide and the central sequence or node is the centroid. The centroid is always the most abundant sequence of the cluster. For sequence comparisons (eg taxonomy) the centroid was used as the representative sequence and is referred to as the OTU. A fasta file of the 1260 most abundant OTU DNA sequences (swarms) is available at Figshare (Digital Object Identifier = 10.6084/m9.figshare.16689487) along with the MiSeq sequence files.

### Statistical analysis and plotting

Matrices of samples by OTU or taxon counts were constructed using python version 2.7. All statistical analyses such as principal components (PCA), differential abundance, correlations (Spearman Rank Correlation) and the plotting of bar and volcano plots, PCA and correlograms were carried out using the R (ver 3.6) libraries Cairo, vegan, fossil, permute, lattice, latticeExtra, colorspace, RColorBrewer, calibrate, ggplot2, corrplot, ggrepel, ggfortify, grid, gridExtra, methods and/or edgeR. All R libraries except edgeR (for differential abundance analysis) were obtained from https://cran.r-project.org. The edgeR library was from https://bioconductor.org/packages/release/bioc/html/edgeR.html. In the edgeR analysis, differential abundance was estimated using the quasi-likelihood F-test with the generalised linear model glmQLFit and glmQLFTest functions recommended by the edgeR authors [24, 25] for differential expression calculations. The resulting fold changes (FC) are expressed as log_2_(FC). For PCA analysis (see below) the cows were assigned to MeY (g CH_4_/kg dry matter intake) groups as follows: low (MeY < 15), medium (15 < MeY < 25) or high (MeY > 25). These groups were based on the mean +/- a standard deviation. This is illustrated in S1B Fig.

## Results

### Methane, 16S amplicons and sequence diversity

**Fig 1:**
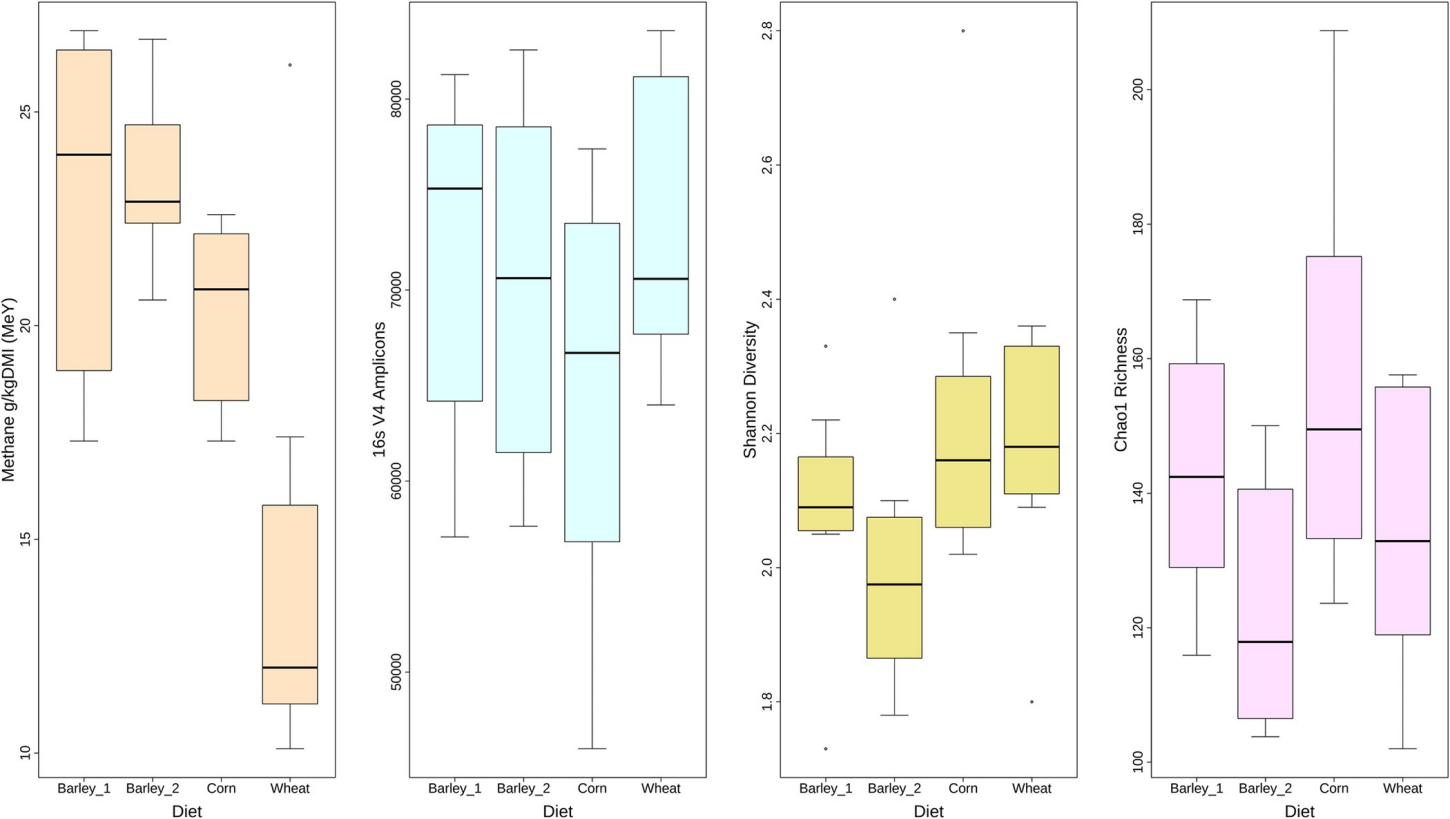
Boxplots illustrating distributions of MeY, 16s amplicon abundance, Shannon diversity and Chao1 richness compared to diet across the herd of 32 cows.

Diet, methane measurements (as methane yield, MeY), 16S amplicon counts (post filtering and paired-end merging), Shannon diversity and Chao1 richness calculations for each cow are summarised as boxplots in Fig 1. Based on a visual inspection of the plot, neither diversity nor richness displayed a tendency for grouping based on diet. The data used for the boxplots in Fig 1 are listed in S1 Table. The number of filtered paired-end-merged 16S rRNA V4 sequences ranged from 45980 to 83571 amplicons per sample. A rarefaction analysis of the amplicon sequences can be seen in S2 Fig. A visual inspection of the figure suggests that in the wheat-fed samples, the number of different Uparse-clustered OTU for a given number of sequences is in general lower than for the barley or corn-fed cows. The Shannon diversity index ranged from 1.73 to 2.8 and the Chao1 richness measure from 102 to 208 (S3 Fig).

### Microbial families versus diet

The initial analysis of taxonomies present in the microbiome rRNA gene V4 amplicon sequences was based on families. Using the Silva 16S rRNA microbial taxonomy database, we found the sequence data from the combined sample collection (32 cows) represented 153 microbial families. At an abundance of at least 100 counts per million (cpm) in at least 5 cows (excluding the unassigned sequences) we found 46 microbial families: 2 archaeal families and 44 bacterial families. When considering the cows as diet-based groups of 8, no group was devoid of any of the 46 microbial families, despite relative abundances varying greatly. The 46 families and their abundances in cpm in each cow are shown in S2A Table.

A core of 19 families (excluding unassigned), all bacterial, no archaeal, were identified in each of the 32 rumen samples at a minimum of 100 cpm abundance. These are indicated with “#” in S2A Table.

**Fig 2:**
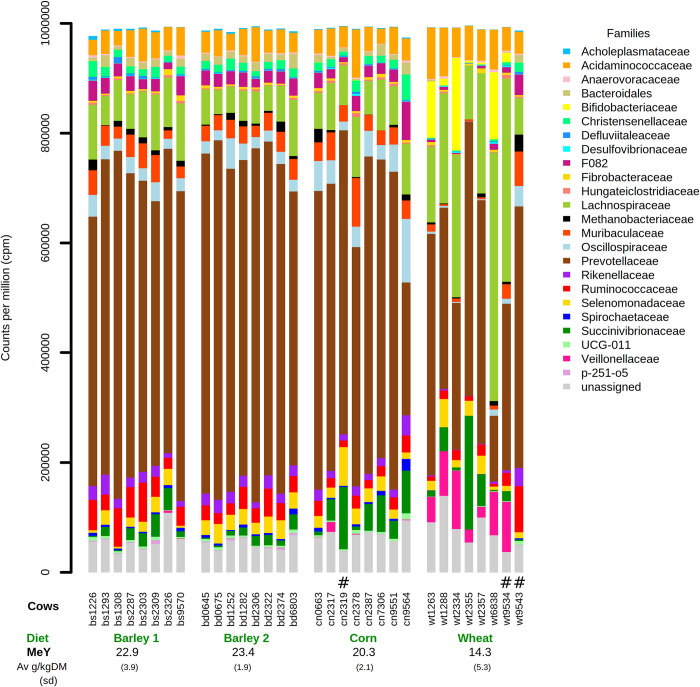
Stacked barplot of the abundance in counts per million (cpm) of the 25 most abundant bacterial plus archaeal families from 32 rumen samples. Also shown is the mean +/- sd CH4 yield (MeY) as gm CH4 per kg DMI for each group of cows and the diet of the group. # indicates cows treated with antibiotics before the feeding trial (Mastalone/wt9543, Yodimaspen/wt9534, CepravinLC/cn2319).

The distribution of the most abundant 24 families (at least 2000cpm in at least 5 cows) is illustrated in the bar chart of Fig 2 (abundances in S2A Table). A basic visual interpretation shows firstly that, at the family level, the ruminal microbiomes of corn and barley-fed dairy cows were almost indistinguishable, being dominated, as expected, by the *Prevotellaceae*. In contrast to the corn and barley-fed cows, in cows fed the wheat based diet, a t-test shows there were substantial differences in abundance of some of the bacterial families (S2B Table). In cows fed the wheat diet, *Acholeplasmataceae*, *Bacteroidales*, *Christensenellaceae*, *Defluviitaleaceae*, *F082*, *Hungateiclostridiaceae*, *Methanobacteriaceae*, *Muribaculaceae*, *Oscillospiraceae*, *p-251-o5*, *Prevotellaceae*, *Rikenellaceae*, *Ruminococcaceae*, *Spirochaetaceae*, *and UCG-011* were less abundant while *Acidaminococcaceae*, *Lachnospiraceae*, *Veillonellaceae* and *Bifidobacteriaceae* were proportionally greater in abundance in wheat-fed compared to corn or barley-fed cows.

### OTU sequences

From the clustered 16S amplicon sequences we identified 7625 clusters or OTU. Of those OTU, 1260 had an abundance of at least 100cpm in at least 5 rumen samples. S3A Table contains a list of the 1260 OTU with their abundance in cpm in each cow’s ruminal microbiome, plus the MeY for each cow. S3B Table contains a list of the same 1260 OTU with their likely taxonomy from the Silva 16s database.

**Fig 3:**
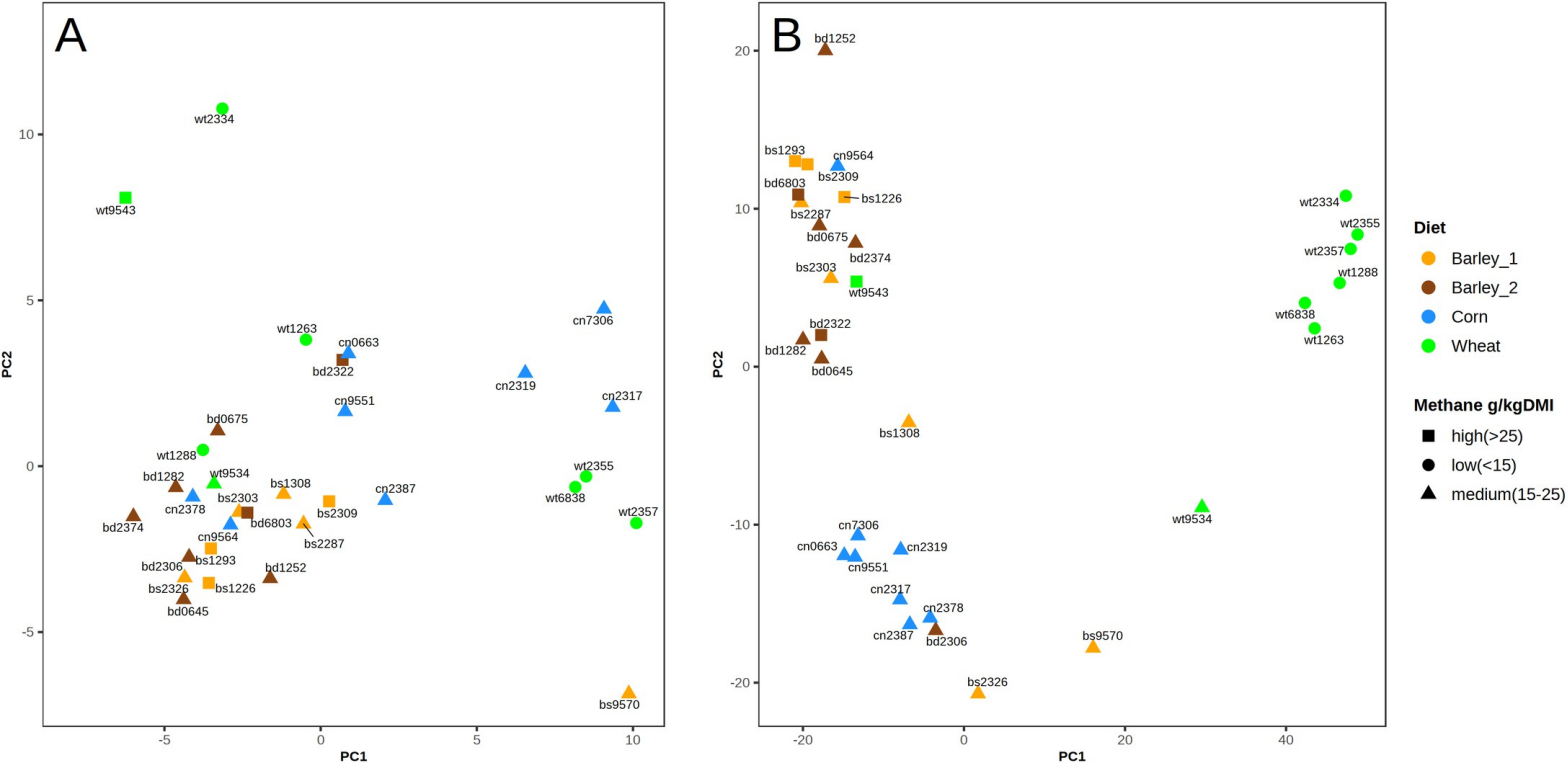
PCA. Principal Components Analysis of rumen sequences using: A: abundance of families derived from unclustered DNA sequences aligned to the Silva 16s taxonomy database, B: abundance of OTU created using the Swarm2 clustering algorithm. For this analysis the cows were assigned to methane yield groups (g CH4/kg dry matter intake, MeY) as follows: low (MeY < 15), medium (15 < MeY < 25) or high (MeY > 25). Also see S1B Fig.

The microbial populations were examined using principal component analysis (PCA), using both the distribution of microbial families and the distribution of OTU from the Swarm clustering. Fig 3A shows a PCA of the families and 3B the OTU. There was marginal clustering visible when basing the analysis on families, but a convincing grouping can be seen of OTU by diet and by MeY. The only two wheat-fed animals (wt9534 & wt9543) that did not cluster with wheat grouping in Fig 3B, and which had higher MeY, had both been treated with antibiotics in the lead up to the experiment. The latter outlier cows were ignored in subsequent analyses. Cow cn2319 was also treated with an antibiotic but did not behave as an outlier in the PCA. This signals that each diet results in a distinct microbial population able to influence MeY. In addition, the PCA confirms there was little if any difference between the two barley diets (single versus double rolled). In further analyses these were treated as a single group of 16 cows.

Of the 1260 OTU present at a minimum 100cpm in 5 cows, a core group of 36 OTU were present in the rumen of every cow (at a minimum 10cpm), although with abundance varying greatly. For example, OTU rs0037 ranged from 29cpm in cow cn2319 to 11854cpm in cow bs1226. Table 1 lists the 36 core OTU with their taxon path as determined by alignment of the DNA sequences versus the Silva 16S rRNA database, using Megablast. Again, *Prevotellaceae* and *Lachnospiraceae* family members made up the bulk of the core OTU and *Methanobacteriaceae* was the only archaeal representative.

**Table 1:** Core OTU.

Core OTU	Taxon path (Silva database)
rs0037	Archaea;Euryarchaeota;Methanobacteria;Methanobacteriales;Methanobacteriaceae;Methanobrevibacter
rs0287	Bacteria;Bacteroidota;Bacteroidia;Bacteroidales;Muribaculaceae
rs0001	Bacteria;Bacteroidota;Bacteroidia;Bacteroidales;Prevotellaceae;Prevotella
rs0002	Bacteria;Bacteroidota;Bacteroidia;Bacteroidales;Prevotellaceae;Prevotella
rs0018	Bacteria;Bacteroidota;Bacteroidia;Bacteroidales;Prevotellaceae;Prevotella
rs0254	Bacteria;Bacteroidota;Bacteroidia;Bacteroidales;Prevotellaceae;Prevotella
rs0009	Bacteria;Bacteroidota;Bacteroidia;Bacteroidales;Prevotellaceae;Prevotella_7
rs0169	Bacteria;Bacteroidota;Bacteroidia;Bacteroidales;Prevotellaceae
rs0236	Bacteria;Bacteroidota;Bacteroidia;Bacteroidales;Prevotellaceae
rs0493	Bacteria;Bacteroidota;Bacteroidia;Bacteroidales;Prevotellaceae
rs0218	Bacteria;Bacteroidota;Bacteroidia;Bacteroidales;Rikenellaceae
rs0386	Bacteria;Bacteroidota;Bacteroidia;Bacteroidales
rs0813	Bacteria;Bacteroidota;Bacteroidia;Bacteroidales
rs0816	Bacteria;Desulfobacterota;Desulfobulbia;Desulfobulbales;Desulfobulbaceae;Desulfobulbus
rs0135	Bacteria;Fibrobacterota;Fibrobacteria;Fibrobacterales;Fibrobacteraceae;Fibrobacter
rs0453	Bacteria;Firmicutes;Clostridia;Lachnospirales;Defluviitaleaceae;Defluviitaleaceae
rs0134	Bacteria;Firmicutes;Clostridia;Lachnospirales;Lachnospiraceae;Lachnoclostridium
rs0040	Bacteria;Firmicutes;Clostridia;Lachnospirales;Lachnospiraceae;Lachnospira
rs0015	Bacteria;Firmicutes;Clostridia;Lachnospirales;Lachnospiraceae
rs0341	Bacteria;Firmicutes;Clostridia;Lachnospirales;Lachnospiraceae
rs0704	Bacteria;Firmicutes;Clostridia;Lachnospirales;Lachnospiraceae
rs0725	Bacteria;Firmicutes;Clostridia;Lachnospirales;Lachnospiraceae
rs0939	Bacteria;Firmicutes;Clostridia;Lachnospirales;Lachnospiraceae
rs0582	Bacteria;Firmicutes;Clostridia;Lachnospirales;Lachnospiraceae;Oribacterium
rs0110	Bacteria;Firmicutes;Clostridia;Lachnospirales;Lachnospiraceae;Pseudobutyrivibrio
rs0051	Bacteria;Firmicutes;Clostridia;Lachnospirales;Lachnospiraceae
rs0028	Bacteria;Firmicutes;Clostridia;Oscillospirales;Oscillospiraceae;NK4A214
rs0600	Bacteria;Firmicutes;Clostridia;Oscillospirales;Oscillospiraceae;NK4A214
rs0467	Bacteria;Firmicutes;Clostridia;Oscillospirales;Oscillospiraceae
rs0226	Bacteria;Firmicutes;Clostridia;Oscillospirales;Ruminococcaceae;Ruminococcus
rs0249	Bacteria;Firmicutes;Clostridia;Oscillospirales;Ruminococcaceae;Ruminococcus
rs0527	Bacteria;Firmicutes;Clostridia;Peptostreptococcales-Tissierellales;Anaerovoracaceae
rs0030	Bacteria;Firmicutes;Negativicutes;Acidaminococcales;Acidaminococcaceae;Succiniclasticum
rs0388	Bacteria;Firmicutes;Negativicutes;Acidaminococcales;Acidaminococcaceae;Succiniclasticum
rs0004	Bacteria;Proteobacteria;Gammaproteobacteria;Enterobacterales;Succinivibrionaceae
rs0681	Bacteria;Synergistota;Synergistia;Synergistales;Synergistaceae;Pyramidobacter

The core OTU present in the rumen of every cow (at a minimum 10cpm), based on alignment to the Silva 16S database, sorted by taxon path.

### Differential OTU abundance versus diet and methane yield

Differential abundance of OTU when contrasting diets, carried out using the edgeR library of R, identified many OTU detectable only when a specific diet was fed to the cows. Note that cows wt9543 and wt9534 were identified as outliers in the PCA and were ignored in this and subsequent analyses. The results from edgeR analysis can be visualised using a volcano plot (log_2_ fold change vs negative log_10_ *P*-value scatter plot). Contrasting the different grains with each other: wheat vs barley, wheat vs corn and barley vs corn identified many OTU abundance differences. These can be seen in S4A Fig. Wheat noticeably induces many differences in OTU abundance when compared to barley or corn, yet there are very few OTU abundance differences seen when contrasting corn and barley with each other.

Here we focussed on contrasting wheat vs corn plus barley grouped together as non-wheat.

**Fig 4:**
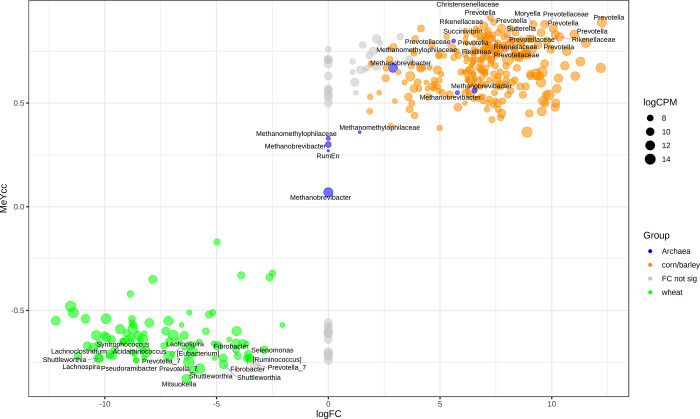
Differential abundance of OTU: Non-wheat vs wheat + MeY. Volcano scatter plot of the result of the Spearman Rank Correlation vs the edgeR differential abundance analysis contrasting wheat diet rumen OTU vs non-wheat. Y axis = methane yield (MeY) correlation, X axis = log2 fold change (logFC). Shown are the bacterial OTU where the *P*-value for the MeY correlation was less than 0.001 and mean abundance at least 128cpm. All archaeal OTU are shown. Grey discs represent OTU where the abundance change between diets was not significant (false discovery rate > 0.02). For ease of visualisation genus labels are shown only for OTU near the extremities of the plot or to highlight certain taxa such as the archaea.

To compare MeY correlations with diet preferences we have replaced the negative log_10_ *P*-value of the standard volcano plot Y axis (see S4A Fig) with the MeY Spearman Rank correlation coefficient (S4A Table with p-value in 4b), creating a plot of MeY correlation versus diet-induced OTU abundance log_2_ fold change (Fig 4), contrasting wheat with corn plus barley (ie non-wheat). The same figure but with OTU labels instead of Genera can be seen in S4B Fig.

There were 395 OTU with mean abundance at least 128cpm and a significant positive or negative correlation with methane abundance (*P* < 0.01). We used these latter bacterial OTU, plus all the archaea-derived OTU, as input to the edgeR differential abundance analysis to identify wheat-linked OTU that are also linked to suppression of methane. Several OTU from the *Acidaminococcus*, *Fibrobacter*, *Lachnoclostridium*, *Lachnospira*, *Mitsuokella*, *Prevotella_7*, *Pseudoramibacter*, *Selenomonas*, *Shuttleworthia* and *Syntrophococcus* genera were significantly enhanced in the wheat-fed rumen microbiome and some correlate strongly with reduced methane, when compared to the non-wheat-fed rumen (Fig 4). Table 2 contains a list of the 25 OTU with the highest fold change in abundance in the wheat-fed rumen samples when comparing them to non-wheat diets. In the reverse direction, genera with high abundance in the corn or barley diets plus high positive MeY correlation include the *Christensenellaceae*, *Flexilinea*, *Moryella*, *Prevotella*, *Prevotellaceae*, *Rikenellaceae*, *Sutterella* plus unidentifiable members of the *Rikenellaceae*, *Lachnospiraceae* and *Muribaculaceae* families. Finally, the methanogens also have an important presence in these microbiomes. Several members of the *Methanobacteriaceae* and *Methanomethylophilaceae* families were increased in abundance in the non-wheat relative to the wheat-fed microbiome, while others of these families showed no difference between wheat and non-wheat diets.

**Table 2:** Wheat-preferring OTU.

OTU	MeY correlation	Log_2_ Fold Change (wheat vs non-wheat)	Family	Genus	% sequence ID
rs0266	-0.72	-11.1987445566688	unknown		
rs0109	-0.73	-10.293510392806	Lachnospiraceae	Shuttleworthia	99.605
rs0179	-0.73	-10.2436331436042	Lachnospiraceae	Lachnospira	97.233
rs0564	-0.72	-9.79733361009569	Lachnospiraceae	Lachnoclostridium	98.024
rs0190	-0.71	-9.76253547779212	Lachnospiraceae	Syntrophococcus	100.000
rs0478	-0.72	-9.62309489077136	unknown		
rs0899	-0.71	-8.6573137807691	unknown		
rs0950	-0.74	-8.6075065092708	Acidaminococcaceae	Acidaminococcus	100.000
rs0784	-0.74	-8.59876443797461	Eubacteriaceae	Pseudoramibacter	100.000
rs0777	-0.73	-8.23420843417952	Prevotellaceae	unknown	99.605
rs0031	-0.72	-7.15201555381884	Prevotellaceae	Prevotella_7	94.862
rs0722	-0.71	-6.97220272343484	Lachnospiraceae	Lachnospira	96.838
rs0090	-0.83	-6.35217377056664	Selenomonadaceae	Mitsuokella	100.000
rs1163	-0.73	-6.33492428890076	Selenomonadaceae		98.413
rs0009	-0.75	-6.24422300111874	Prevotellaceae	Prevotella_7	100.000
rs0215	-0.79	-6.01831007436096	Selenomonadaceae		100.000
rs0054	-0.78	-5.74055437739996	Lachnospiraceae	Shuttleworthia	100.000
rs0984	-0.73	-5.09755582544947	Anaerovoracaceae	[Eubacterium]	100.000
rs0192	-0.73	-4.71167445103454	Selenomonadaceae		100.000
rs0111	-0.76	-4.68726589983386	Lachnospiraceae	Shuttleworthia	100.000
rs0315	-0.72	-4.05059043488553	Fibrobacteraceae	Fibrobacter	100.000
rs0927	-0.73	-3.62566978091586	unknown		
rs0141	-0.73	-3.62091371180705	Fibrobacteraceae	Fibrobacter	100.000
rs0301	-0.71	-3.58312346950369	Selenomonadaceae	Selenomonas	100.000
rs0149	-0.73	-3.50557978152095	Lachnospiraceae	[Ruminococcus]	99.605

The 25 OTU found in the wheat-fed cows with the greatest change in abundance compared to corn or barley-fed cows, sorted by log_2_ fold change. Sequence identity (ID) is based on alignment with 16S sequences in the Silva database.

### Methanogens

The sequence clustering yielded 9 OTU from methanogenic archaea, belonging to the *Methanobacteriaceae* and *Methanomethylophilaceae* families. Two OTU dominated the archaeal population. Using the Genbank 16S rRNA database to obtain likely genus and species-level taxa, *Methanobrevibacter millerae* (rs0036, 99.6% sequence identity) was present more in the barley or corn-fed cows compared to wheat. In contrast, *Methanobrevibacter olleyae* (rs0037, 99.2% sequence identity) was present in all cows and its abundance had no significant correlation with MeY. The correlation analysis versus MeY gave rs0036 a moderate positive coefficient of 0.67(*P* = 0.001), while that of rs0037 is only 0.07 (*P* = 0.5). A one-tailed t-test confirms rs0036 abundance was greater in the rumen of corn or barley-fed cows than in cows fed wheat (see S5 Table).

### OTU correlations versus MeY, pH and other fermentation products

**Fig 5:**
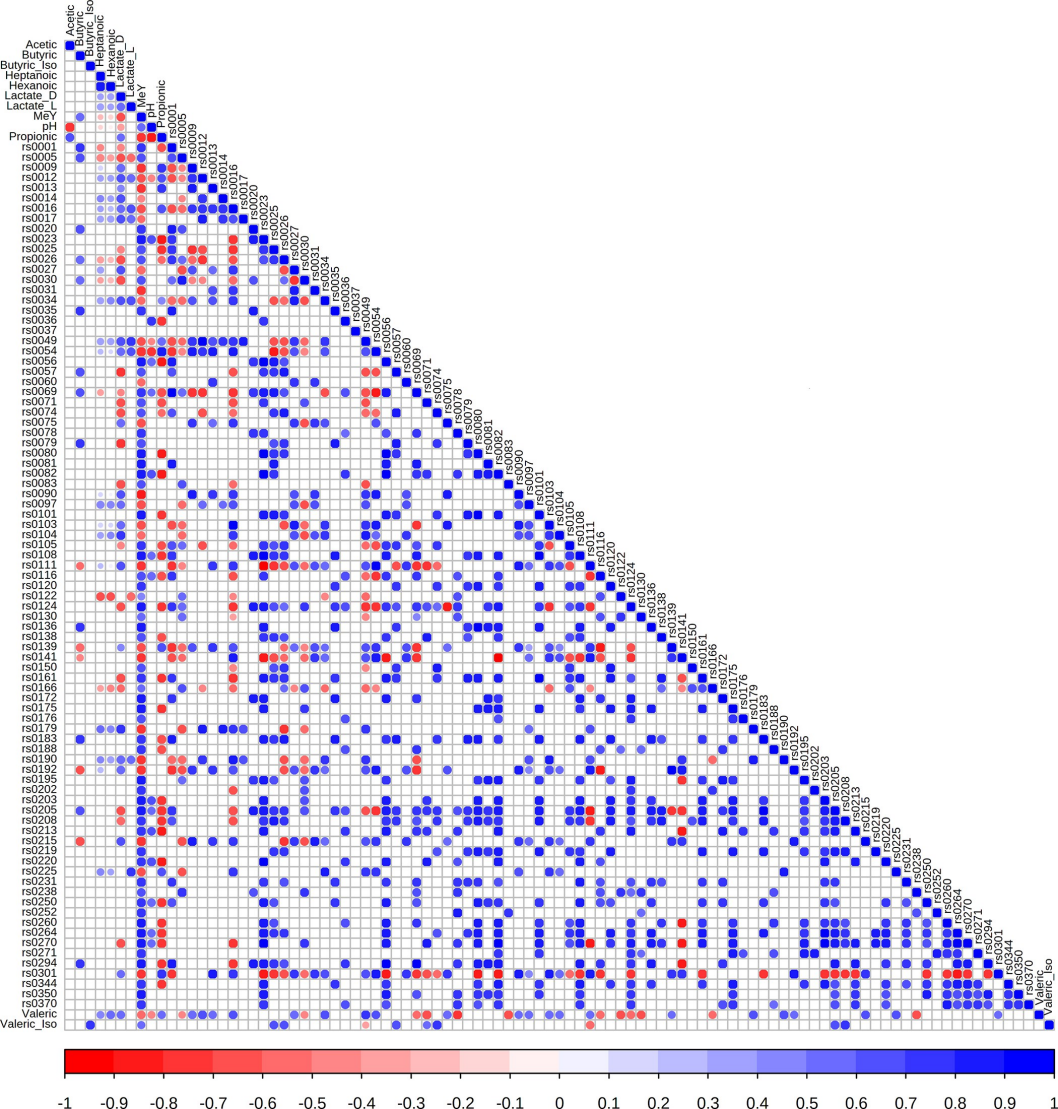
Correlation of OTU vs MeY, pH and ruminal acids. Correlogram illustrating the Spearman Rank Correlation analysis of the abundance of 83 OTU versus each other and MeY (CH4 yield), ruminal pH, D-lactate, L-lactate, acetic acid, propionic acid, iso-butyric acid, butyric acid, iso-valeric acid, valeric acid, hexanoic acid and heptanoic acid. The 83 OTU are those with an abundance of at least 1000cpm in at least 5 cows, a significant correlation with MeY (*P*<0.01) and a logFC (log2 fold change) magnitude of at least 2.5 when comparing a wheat to a non-wheat diet. Also included are the 2 most abundant methanogenic archaea. Correlation coefficients are rendered as coloured circles (Red negative or Blue positive) if *P*<0.001. Colour intensity reflects degree of correlation.

Spearman Rank Correlation analysis of 1260 OTU (those with abundance at least 100cpm in at least 5 cows) in comparison to MeY levels identified 159 OTU with a positive MeY correlation greater than or equal to 0.7 and 31 OTU with a negative MeY correlation less than or equal to -0.7. The 83 OTU with an MeY correlation *P*-value less than 0.001, a logFC (differential abundance, wheat vs non-wheat) magnitude greater than 2.5 and an OTU abundance of 1000cpm in at least 5 cows were analysed for correlations with MeY, pH, NH4, D-lactate, L-lactate and acetic, propionic, iso-butyric, butyric, iso-valeric, valeric, hexanoic and heptanoic acids (S6 Table). The methanogen OTU rs0036 and rs0037 were included. The correlation coefficients can be seen plotted as a correlogram in Fig 5.

Correlation results for the methanogens plus the 20 OTU with the most negative (ie most anti-methane) MeY correlation are listed in Table 3 (details in S4A and S4B Table), together with pH, lactate and those for acetic and propionic acids and their taxonomic assignments from the Silva database. Other factors analysed yielded only weak correlations.

**Table 3:** Correlation of OTU vs ruminal MeY, pH and volatile fatty acids (VFA).

	Correlation coefficients						logFC	%ID	Taxon
	MeY	pH	D-lactate	L-lactate	Acetic	Propionic			
**MeY**		0.55	-0.61	-0.58	-0.38	-0.76			
**pH**	0.55		-0.4	-0.41	-0.8	-0.89			
**rs0036**	0.67	0.72	-0.39	-0.45	-0.48	-0.76	2.89	99.61	Methanobrevibacter millerae
**rs0037**	0.07	0.41	0.15	-0.21	-0.46	-0.26	0	99.21	Methanobrevibacter olleyae
**rs0090**	-0.83	-0.6	0.6	0.61	0.34	0.8	-6.35	100.0	Mitsuokella jalaludinii
**rs0215**	-0.79	-0.42	0.63	0.51	0.25	0.65	-6.01	100.0	Selenomonadaceae
**rs0054**	-0.78	-0.7	0.57	0.61	0.41	0.88	-5.74	100.0	Shuttleworthia
**rs0111**	-0.76	-0.68	0.62	0.57	0.39	0.86	-4.68	100.0	Shuttleworthia
**rs0009**	-0.75	-0.51	0.59	0.57	0.29	0.73	-6.24	100.0	Prevotella_7
**rs0141**	-0.73	-0.54	0.48	0.56	0.3	0.76	-3.62	100.0	Fibrobacter
**rs0179**	-0.73	-0.4	0.74	0.61	0.24	0.56	-10.24	97.2	Lachnospira
**rs0192**	-0.73	-0.61	0.59	0.5	0.35	0.79	-4.71	99.2	Selenomonas bovis
**rs0031**	-0.72	-0.29	0.53	0.63	0.23	0.53	-7.15	94.8	Prevotella_7
**rs0190**	-0.71	-0.39	0.6	0.56	0.26	0.59	-9.76	100.0	Syntrophococcus
**rs0301**	-0.71	-0.59	0.53	0.55	0.39	0.77	-3.58	100.0	Selenomonas
**rs0013**	-0.7	-0.51	0.46	0.57	0.3	0.71	-6.08	100.0	Prevotella_7
**rs0097**	-0.68	-0.23	0.55	0.63	0.19	0.38	-10.52	94.4	Shuttleworthia
**rs0103**	-0.67	-0.39	0.57	0.57	0.16	0.65	-6.18	100.0	Prevotella_7
**rs0075**	-0.66	-0.37	0.6	0.63	0.28	0.57	-4.12	100.0	Prevotella_7
**rs0139**	-0.66	-0.49	0.44	0.54	0.34	0.68	-3.82	100.0	Selenomonadaceae
**rs0016**	-0.64	-0.41	0.56	0.65	0.23	0.63	-8.30	100.0	Succiniclasticum
**rs0225**	-0.63	-0.45	0.56	0.72	0.37	0.61	-8.82	96.0	Selenomonadaceae
**rs0012**	-0.62	-0.46	0.61	0.58	0.33	0.61	-6.94	99.6	Dialister succinatiphilus
**rs0049**	-0.62	-0.42	0.67	0.73	0.34	0.58	-10.38	100.0	Acidaminococcus fermentans

The Spearman Rank correlation coefficients of MeY, pH, the 2 most abundant methanogen OTU and the 20 OTU with the strongest MeY correlations versus MeY, ruminal pH, lactate and acetic and propionic acids plus their taxa (Genus or Family, with species where available at the indicated percent sequence identity) as identified using the Silva 16S taxonomy database or the Genbank 16S database for species level. Also shown is the fold change (log_2_) in abundance of the OTU in the wheat-based versus non-wheat diet identified by edgeR (negative = wheat-preferred, positive = non-wheat-preferred).

The OTU rs0036, most likely from *Methanobrevibacter millerae*, had a strong correlation with MeY and pH plus a strong negative correlation with propionic acid. However, the OTU rs0037, most likely from *Methanobrevibacter olleyae*, had almost no correlation with MeY and no more than a weak correlation with any of the other factors measured. Of the bacteria, rs0090, most likely from *Mitsuokella jalaludinii*, had the strongest negative correlation, -0.83, with MeY. This OTU had a moderate negative correlation with pH together with a moderate positive correlation with lactate and a strong correlation with propionic acid. As the logFC suggests, OTU rs0090 is predominantly found in the wheat-fed rumen. Following on from rs0090, the OTU (rs0054, rs0111 and rs0215) from the *Shuttleworthia* genus and *Selenomonadaceae* family also had strong negative correlations with MeY, negative correlations with pH and positive correlations with lactate and propionic acid. Note that OTU rs0215, from the *Selenomonadaceae* family, of unknown genus or species, but having 98% sequence identity with *Mitsuokella jalaludinii* 16S rRNA V4, had a strong negative correlation with MeY but only a weak negative correlation with pH. OTU rs0109, also from *Shuttleworthia* has a strong correlation with MeY and is strongly associated with wheat, but is of lower abundance and so does not appear in this figure. More details can be seen in Table 3 and S4A and S4B Table. MeY itself had only a weak positive correlation with ruminal pH, but a moderate negative correlation with lactate and a strong negative correlation with propionic acid. Ruminal pH had strong negative correlations with acetic and propionic acids.

### Antibiotics

S7 Table shows the results of z-score calculations to discover which OTU were depressed in cows administered antibiotics for treatment of mastitis prior to the trial ((Mastalone/wt9543, Yodimaspen/wt9534, CepravinLC/cn2319). Among those OTU with a z-score less than -1 are the OTU with the strongest MeY and pH negative correlations. In the Mastalone-treated, wheat-fed cow, wt9543, the 2 members of the *Shuttleworthia* genus (OTU rs0054 and rs0111) were very much depressed in abundance (1.8 and 2 standard deviations below the mean of the other wheat-fed cows). These 2 OTU have the strongest negative correlations with ruminal pH (-0.7 and -0.68 respectively). The ruminal pH itself in this cow had increased by more than 2 standard deviations from the mean of the pH in the other wheat-fed cows (from a mean of 5.89 to a pH of 7.14). The OTU rs0090, from *Mitsuokella* and having the strongest negative correlation with MeY (-0.83), was also depressed by Mastalone, by 1 standard deviation from the mean. In the Mastalone-treated cow MeY increased by over 10 standard deviations above the mean for the wheat-fed group.

## Discussion

The first port of call when analysing the impact of a dietary treatment on the rumen microbiome is usually the archaeal methanogen population. While several different families of archaea were seen, mostly at low abundance, by far the most abundant were 2 members of the *Methanobrevibacter* genus. Based on a more than 99% 16S rRNA V4 sequence identity according to the Genbank 16S database, these were likely *M*. *millerae* and *M*. *olleyae*. These 2 species behaved differently. On average, the abundance of *M*. *olleyae* changed little from one diet to another, whereas *M*. *millerae* tended to be less abundant in the wheat-fed versus the corn or barley-fed cows. Coinciding with the difference in abundance, the OTU rs0036, from *M*. *millerae*, had a strong correlation with MeY and ruminal pH, consistent with previous studies [26] suggesting a link between low pH, *M*. *millerae* abundance and reduced methane yield. *M*. *millerae* abundance, MeY and pH all had very strong negative correlations with the level of propionic acid, but it is not clear whether propionic acid has a suppressing effect on MeY because of its acidity or because its production results in reduced hydrogen availability.

This feeding trial provided us with fortuitous observations which may help elucidate possible associations between the use of antibiotics and effects on the rumen microbiome. The impact of antibiotic treatment on methane production and rumen microbiome makeup of the wheat-fed cows is important. While not carried out here as a deliberate experiment it suggests that key bacterial species, which would otherwise trigger a suppression of methanogen activity, are sensitive to such antibiotic treatment. The fact that the OTU with the greatest sensitivity to the antibiotics used also includes those with the highest correlation to MeY and ruminal pH means that in the future, with properly replicated experiments, antibiotics may constitute a valuable tool for dissecting the microbial population and identifying those key species.

The microbiome changes we observed when analysing bacterial and archaeal families in wheat-fed compared to corn or barley-fed cows is consistent with our hypothesis that microbial species changes in the wheat-fed rumen would explain the lowered MeY seen in those cows compared to the corn or barley-fed cows. Further analysis at the OTU level confirms the hypothesis. The principal component analysis (PCA), differential abundance and correlation analyses of 16S rRNA-based OTU abundances confirm that significant differences exist between the microbiomes of cows fed different grain-supplemented diets and that those microbial population changes are closely linked to MeY. This confirms our hypothesis regarding microbial species found predominantly in the rumen of wheat-fed cows being associated with a reduction in MeY. We found 86 OTU (representing different species or strains) with a very high fold change in abundance (more than 12 times higher in wheat than in corn or barley-fed cows). Of the latter, 25 were also strongly correlated to a decrease in MeY (Spearman Rank correlation below -0.7). High on the list of genera linked to the wheat-based diet and lower MeY, were genera such as the saccharolytic *Shuttleworthia* [27] and *Prevotella_7* [28], associated with production of butyrate, acetate and lactate. Top of the list of wheat-preferring, anti-methane OTU, rs0090, likely representing the rumen bacterium *Mitsuokella jalaludinii* (100% 16S rRNA V4 sequence identity), from the *Selenomonadaceae* family, had the highest correlation (-0.83) with a reduction in MeY. *M*. *jalaludinii* is known to produce lactic and acetic acids [29], contributing to a lowering of pH, but more importantly it is able to reduce fumarate to succinate, which in turn can be reduced to propionate [30]. Interestingly, we found MeY had a stronger negative correlation to propionic acid than to either lactate, acetate or even to ruminal pH. The reduction reactions known to occur in *M*. *jalaludinii* would deprive the methanogenic archaea of hydrogen, a vital ingredient for methane production. Indeed, others have already shown the value of *M*. *jalaludinii* as a potential probiotic bacterial species for methane mitigation in an *in vitro* rumen fermentation system [31]. Other bacteria preferencing the wheat-supplemented diet, represented by OTU such as rs0215 from the *Selenomonadaceae* family and some also from the genera *Prevotella_7*, *Fibrobacter* and *Syntrophococcus* with high anti-methane correlation but low correlation to acidity, need more detailed molecular and functional analysis and likely represent important anti-methanogenic species with potential as probiotic treatments.

In conclusion we propose that wheat, as fed to dairy cows in our feeding trial, has as yet undefined properties which promote the growth of several different ruminal bacterial species seen only at very low abundance in cows fed other grains, such as corn or barley. The impact of these species is two-fold: firstly ruminal pH is lowered, likely restricting growth of one of the main methanogenic archaea (*Methanobrevibacter millerae*) and hence contributing to a lowering of MeY; secondly, and possibly of more importance in terms of MeY, growth of the rumen fumarate-reducing bacterium *Mitsuokella jalaludinii* is promoted, reducing the availability of hydrogen for the generation of methane and in the process, producing acetate, lactate and possibly propionate, contributing to ruminal acidity. Those OTU from species with high anti-methane correlation but low correlation to acidity, and hence a lowered acidosis risk, represent ideal candidate anti-methane probiotic species.

## Supporting information

S1 Fig Timeline of the feeding trial. **a.** CRN (rolled corn), WHT (rolled wheat), SRB (single-rolled barley), DRB (double-rolled barley). **b.** Boxplot distribution of cow MeY measurements overlaid with mean +/- standard deviation positions for grouping into high, low and medium MeY for PCA.
(TIF)

S2 Fig Rarefaction plot of MiSeq amplicon sequences: Number of Uparse 97% sequence identity clusters for increasing number of sequence reads. Legend colours refer to cows.
(TIF)

S3 Fig Scatter plot showing log_2_ of the Shannon diversity and Chao1 richness for each cow (ie each rumen sample). (TIF)

S4 Fig Volcano scatter plots showing the results, using edgeR, of contrasting OTU abundance from the rumen of cows fed different diets (log_2_ fold change vs negative log_10_ *P*-value scatter plot). **a.** A = wheat vs barley, B = wheat vs corn, C = barley vs corn, D = wheat vs non-wheat. OTU labels are shown for OTU where the magnitude of the log2 fold change (logFC) is greater than 7. **b.** Same as Fig 4, but with OTU labels instead of genus.
(TIF)

S1 Table List of cows showing diet, methane, sequence count, Shannon diversity and Chao1 richness. (DOCX)

S2 Table **a.** The 46 families with abundance at least 100cpm in at least 5 cows, showing abundance in cpm for each family (using Silva 16s taxonomy database) in each cow. Note there are 33 columns in this table. # indicates core families. **b.** Welch t-test results for Family abundance comparisons.
(ZIP)

S3 Table **a.** OTU abundances per cow where abundance is >100cpm in at least 5 cows (1260 OTU). Note table is comma-delimited. **b.** OTU taxonomies determined using BLAST vs the Silva 16s rRNA database.
(ZIP)

S4 Table **a.** Spearman Rank Correlation Coefficients. Note table is comma delimited. **b.** Spearman Rank Correlation p values. Note table is comma delimited.
(ZIP)

S5 Table 1-tailed t-test of methanogen abundance to test if wheat vs non-wheat are different. (DOCX)

S6 Table List of cows versus diet and rumen variables pH, ammonia, volatile fatty acids (mg/L). Note table is comma delimited.
(CSV)

S7 Table Z-score analysis to determine which OTU were suppressed by antibiotics. Mastalone (cow wt9543), Yodimaspen (cow9534) or CepravinLC/cn2319). The list includes rs0111, rs0054, rs0090 and rs0215. These were in the top 10 OTU having the strongest anti-methane correlations.
(DOCX)
