# Supplementary figures and images for: Dietary wheat and reduced methane yield are linked to rumen microbiome changes in dairy cows
Source: PLoS One. 2022 May 19;17(5):e0268157. doi: 10.1371/journal.pone.0268157 (PMC9119556; doi:10.1371/journal.pone.0268157)

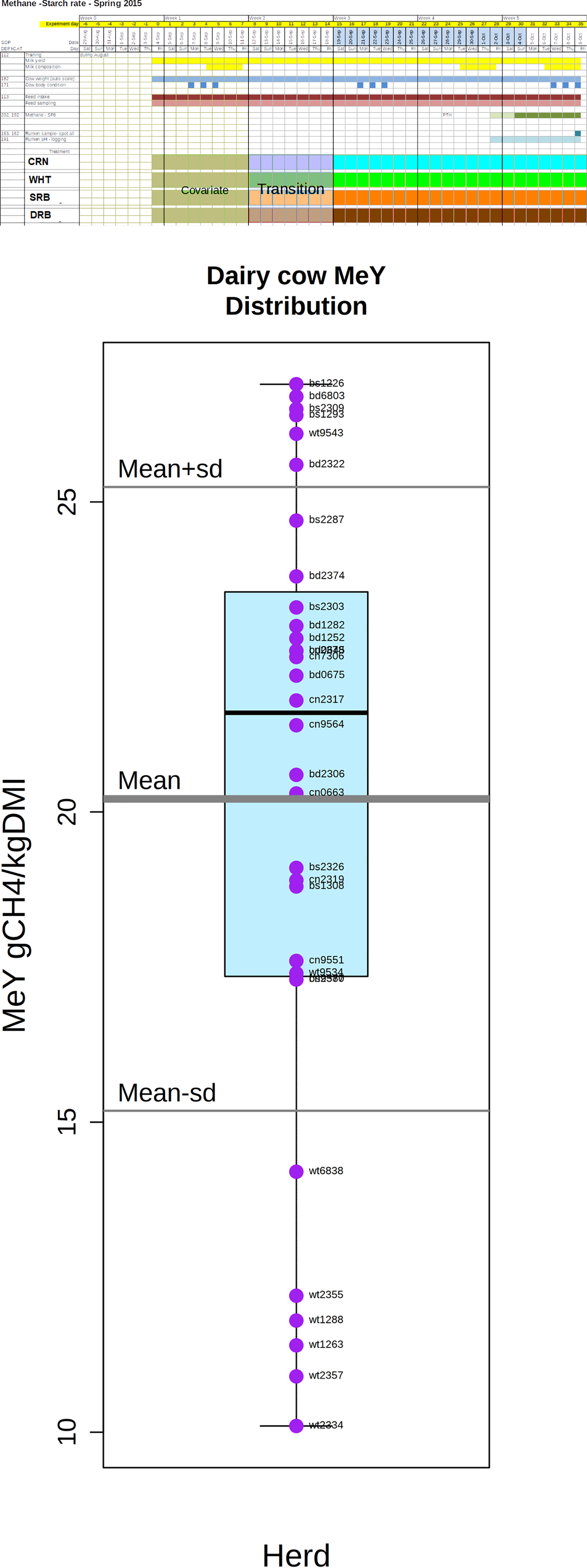

Supplement: S1 Fig — a. CRN (rolled corn), WHT (rolled wheat), SRB (single-rolled barley), DRB (double-rolled barley). b. Boxplot distribution of cow MeY measurements overlaid with mean +/- standard deviation positions for grouping into high, low and medium MeY for PCA. (TIF) [file pone.0268157.s001.tif]

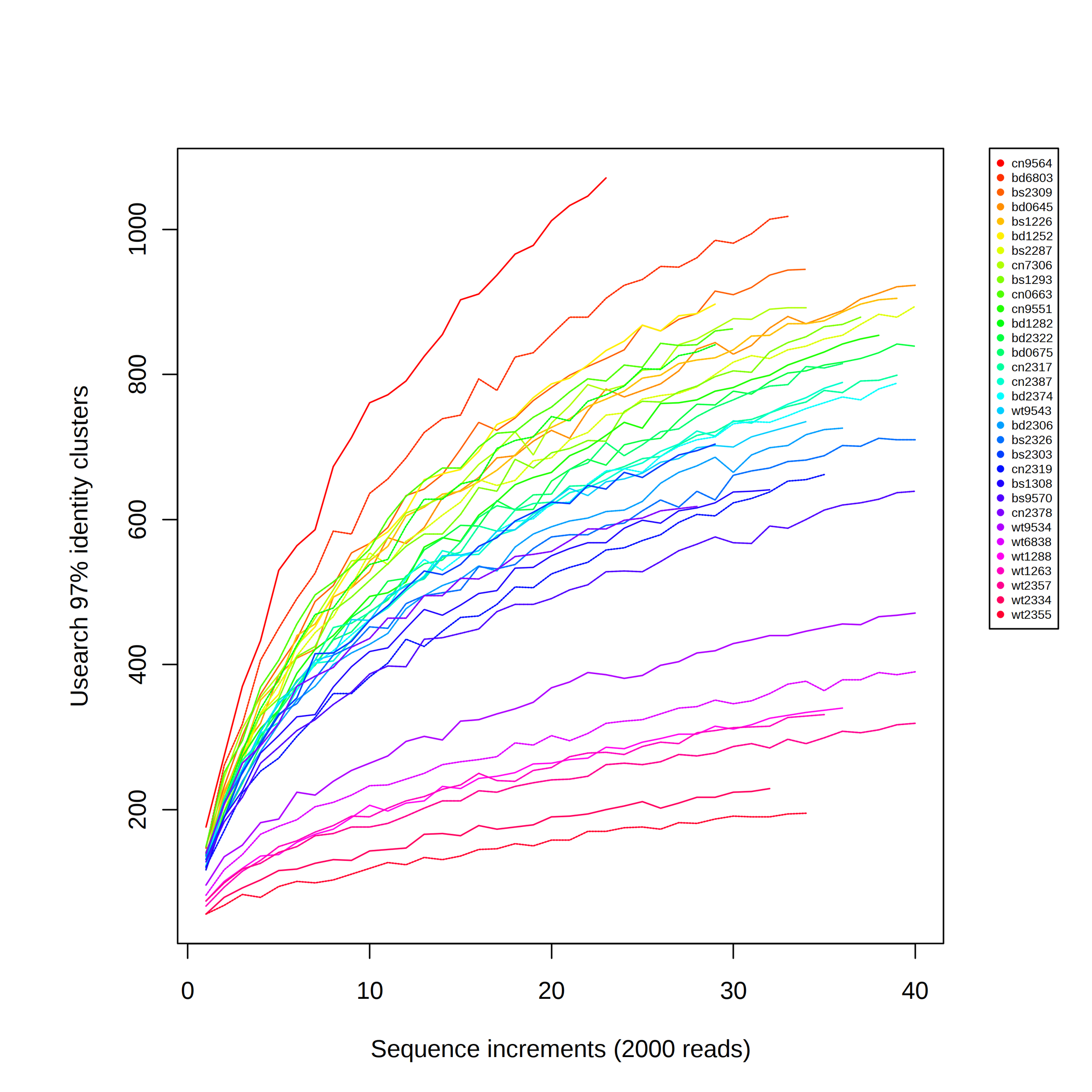

Supplement: S2 Fig — Legend colours refer to cows. (TIF) [file pone.0268157.s002.tif]

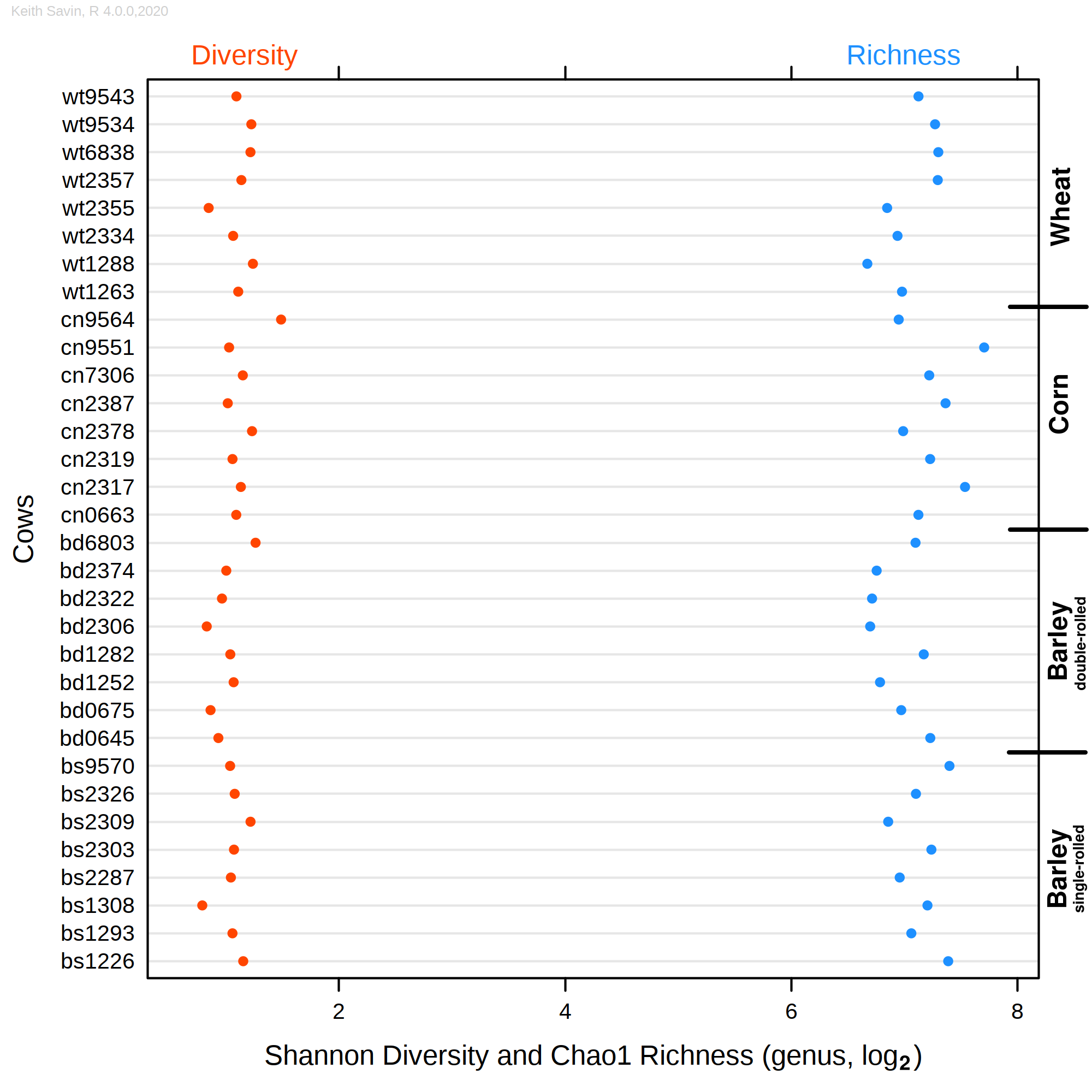

Supplement: S3 Fig — (TIF) [file pone.0268157.s003.tif]

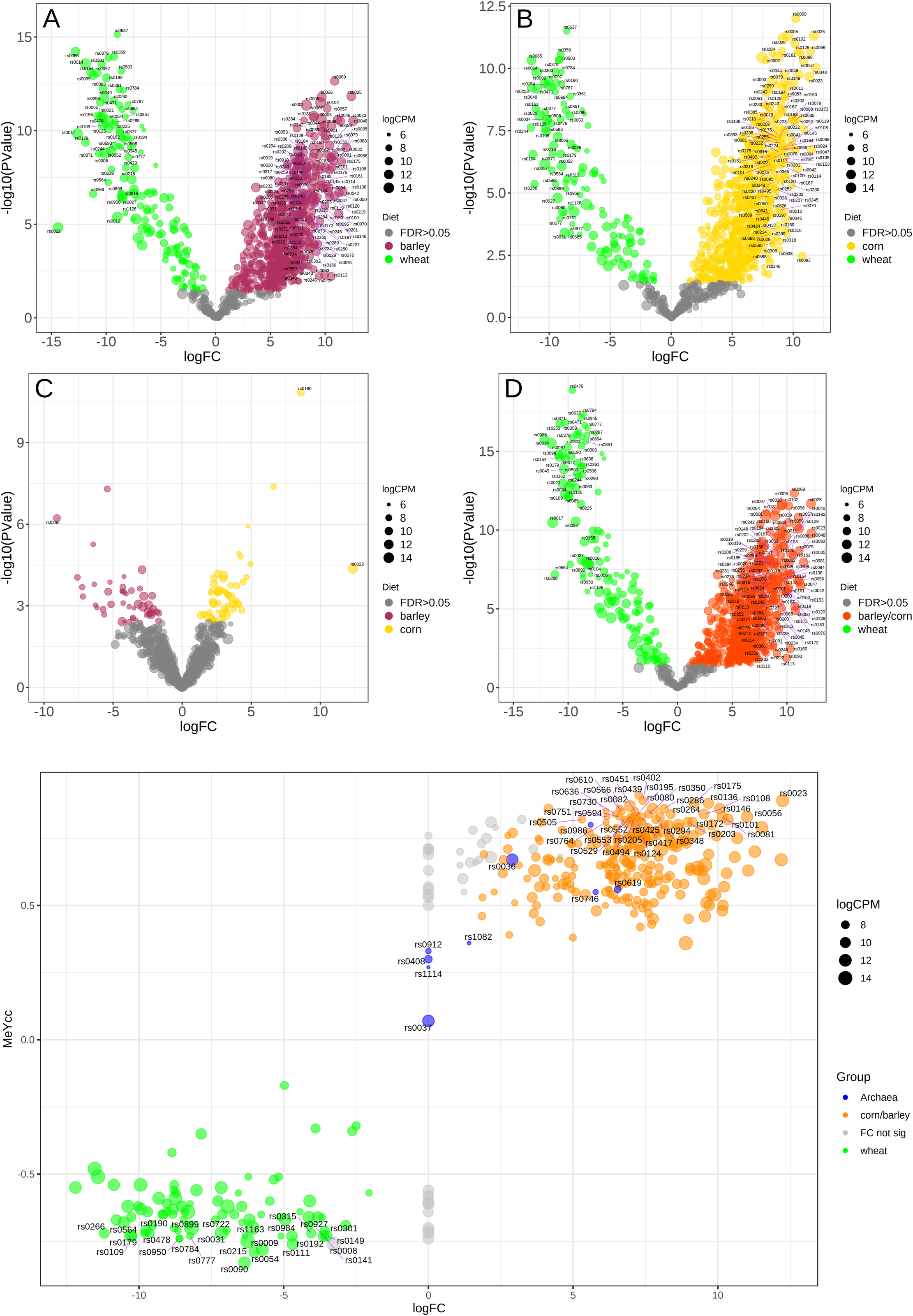

Supplement: S4 Fig — a. A = wheat vs barley, B = wheat vs corn, C = barley vs corn, D = wheat vs non-wheat. OTU labels are shown for OTU where the magnitude of the log2 fold change (logFC) is greater than 7. b. Same as Fig 4, but with OTU labels instead of genus. (TIF) [file pone.0268157.s004.tif]
