# Supplementary material for: Dietary wheat and reduced methane yield are linked to rumen microbiome changes in dairy cows
Source: PLoS One. 2022 May 19;17(5):e0268157. doi: 10.1371/journal.pone.0268157 (PMC9119556; doi:10.1371/journal.pone.0268157)
Supplement: S1 Table — (DOCX) [file pone.0268157.s005.docx]

| **Cow** | **Diet** | **MeY** | **amplicons** | **Shannon** | **Chao1** |
| --- | --- | --- | --- | --- | --- |
| bs1226 | Barley (single rolled) | 26.9 | 77270 | 2.22 | 167.45 |
| bs1293 | Barley (single rolled) | 26.4 | 74172 | 2.08 | 133.57 |
| bs1308 | Barley (single rolled) | 18.8 | 62158 | 1.73 | 147.45 |
| bs2287 | Barley (single rolled) | 24.7 | 80011 | 2.06 | 124.38 |
| bs2303 | Barley (single rolled) | 23.3 | 57077 | 2.10 | 151.05 |
| bs2309 | Barley (single rolled) | 26.5 | 66205 | 2.33 | 115.91 |
| bs2326 | Barley (single rolled) | 19.1 | 76441 | 2.11 | 137.41 |
| bs9570 | Barley (single rolled) | 17.3 | 81277 | 2.05 | 168.75 |
| bd0645 | Barley (double rolled) | 22.6 | 79426 | 1.91 | 150.03 |
| bd0675 | Barley (double rolled) | 22.2 | 71015 | 1.82 | 125.56 |
| bd1252 | Barley (double rolled) | 22.8 | 57631 | 2.10 | 110.25 |
| bd1282 | Barley (double rolled) | 23.0 | 57895 | 2.05 | 144.14 |
| bd2306 | Barley (double rolled) | 20.6 | 70228 | 1.78 | 103.80 |
| bd2322 | Barley (double rolled) | 25.6 | 82567 | 1.95 | 105.00 |
| bd2374 | Barley (double rolled) | 23.8 | 77658 | 2.00 | 108.00 |
| bd6803 | Barley (double rolled) | 26.7 | 65083 | 2.40 | 137.06 |
| cn0663 | Corn (single rolled) | 20.3 | 59098 | 2.13 | 139.50 |
| cn2317 | Corn (single rolled) | 21.8 | 77388 | 2.19 | 185.64 |
| cn2319 | Corn (single rolled) | 18.9 | 69353 | 2.08 | 149.90 |
| cn2378 | Corn (single rolled) | 22.6 | 54540 | 2.35 | 127.05 |
| cn2387 | Corn (single rolled) | 17.3 | 71691 | 2.02 | 164.75 |
| cn7306 | Corn (single rolled) | 22.5 | 64072 | 2.22 | 149.06 |
| cn9551 | Corn (single rolled) | 17.6 | 75293 | 2.04 | 208.75 |
| cn9564 | Corn (single rolled) | 21.4 | 45980 | 2.80 | 123.64 |
| wt1263 | Wheat (single rolled) | 11.4 | 69384 | 2.16 | 126.17 |
| wt1288 | Wheat (single rolled) | 11.8 | 71791 | 2.36 | 102.00 |
| wt2334 | Wheat (single rolled) | 10.1 | 63980 | 2.09 | 122.72 |
| wt2355 | Wheat (single rolled) | 12.2 | 68031 | 1.80 | 115.17 |
| wt2357 | Wheat (single rolled) | 10.9 | 83571 | 2.20 | 157.07 |
| wt6838 | Wheat (single rolled) | 14.2 | 83547 | 2.32 | 157.60 |
| wt9534 | Wheat (single rolled) | 17.4 | 78794 | 2.34 | 154.50 |
| wt9543 | Wheat (single rolled) | 26.1 | 67346 | 2.13 | 139.57 |

Cows from which rumen samples were obtained, showing diet, methane yield (MeY), 16S variable region 4 PCR amplicon sequence count, Shannon diversity and Chao1 species richness.
