# Supplementary material for: Dietary wheat and reduced methane yield are linked to rumen microbiome changes in dairy cows
Source: PLoS One. 2022 May 19;17(5):e0268157. doi: 10.1371/journal.pone.0268157 (PMC9119556; doi:10.1371/journal.pone.0268157)
Supplement: S5 Table — (DOCX) [file pone.0268157.s009.docx]

| **OTU** | **abundance mean_Non-wheat** | **abundance mean_Wheat** | **t** | **df** | **p-value** | **Family** | **Genus** |
| --- | --- | --- | --- | --- | --- | --- | --- |
| rs0036 | 5385.7304 | 509.9733 | 4.5923 | 25.417 | 5.174e-05 | Methanobacteriaceae | Methanobrevibacter |
| rs0037 | 4622.999 | 2985.485 | 1.2717 | 25.091 | 0.1076 | Methanobacteriaceae | Methanobrevibacter |
| rs0408 | 248.78487 | 78.77076 | 2.1094 | 12.287 | 0.02803 | Methanobacteriaceae | Methanobrevibacter |
| rs0619 | 186.2265 | 0.0000 | 2.8906 | 23 | 0.004125 | Methanobacteriaceae | Methanobrevibacter |
| rs0746 | 113.3066 | 0.0000 | 3.1031 | 23 | 0.002505 | Methanobacteriaceae | Methanobrevibacter |
| rs0912 | 88.21227 | 50.20522 | 1.239 | 8.5234 | 0.1242 | Methanomethylophilaceae |  |
| rs0986 | 99.97924 | 0.00000 | 5.1384 | 23 | 1.656e-05 | Methanomethylophilaceae |  |
| rs1082 | 72.56681 | 24.54716 | 2.7102 | 14.364 | 0.008306 | Methanomethylophilaceae |  |
| rs1114 | 65.48700 | 34.73197 | 1.6338 | 9.7444 | 0.06708 | Methanomethylophilaceae | RumEn |

One-tailed t-test analysis of archaeal abundances comparing diets: wheat vs non-wheat

eg For rs0036 t=4.6

The difference between the 2 groups(non-wheat,vs,wheat)

is 4.6 times more than the difference between individuals within each group.

% sequence identity = 100% (Silva database) for each OTU.
