# Supplementary material for: Dietary wheat and reduced methane yield are linked to rumen microbiome changes in dairy cows
Source: PLoS One. 2022 May 19;17(5):e0268157. doi: 10.1371/journal.pone.0268157 (PMC9119556; doi:10.1371/journal.pone.0268157)
Supplement: S7 Table — Mastalone (cow wt9543), Yodimaspen (cow9534) or CepravinLC/cn2319). The list includes rs0111, rs0054, rs0090 and rs0215. These were in the top 10 OTU having the strongest anti-methane correlations. (DOCX) [file pone.0268157.s011.docx]

**z-score calculations for comparison of antibiotic-treated cows**

(Mastalone/wt9543, Yodimaspen/wt9534, CepravinLC/cn2319)

Showing data for OTU where z-score < -1 (OTU abundance down more than 1 standard deviation. cn2319 only 20 most changed OTU are listed out of 170)

**Yodimaspen/wt9534**

OTU count mean variance std_dev wt9543 z-score Family

rs0016 6 52349.4 1.16554e+08 10796 19697.38013 -3.02443 Acidaminococcaceae

rs0257 6 54.7008 1080.74 32.8747 0.00000 -1.65887 F082

rs0301 6 1172.79 202601 450.112 464.37831 -1.57351 Selenomonadaceae

rs0097 6 8908.24 3.51807e+07 5931.33 0.00000 -1.50187 Lachnospiraceae

#rs0111 6 5849.41 8.12613e+06 2850.64 1896.21145 -1.38673 Lachnospiraceae

rs0135 6 98.8334 4256.69 65.2433 12.89940 -1.31511 Fibrobacteraceae

rs0034 6 22703 1.39693e+08 11819.2 7172.06506 -1.31403 unknown

rs0027 6 24973.6 3.13359e+08 17701.9 3779.52350 -1.19727 Prevotellaceae

rs0049 6 16584.9 1.83125e+07 4279.32 11970.64097 -1.07826 Acidaminococcaceae

#rs0090 6 8871.26 6.59626e+07 8121.74 722.36627 -1.00333 Selenomonadaceae

**Mastalone/wt9543**

OTU count mean variance std_dev wt9543 z-score Family

rs0016 6 52349.4 1.16554e+08 10796 0.00000 -4.84892 Acidaminococcaceae

rs0049 6 16584.9 1.83125e+07 4279.32 0.00000 -3.87552 Acidaminococcaceae

rs0301 6 1172.79 202601 450.112 0.00000 -2.60497 Selenomonadaceae

rs0127 6 2242.07 926311 962.45 15.21445 -2.3135 Lachnospiraceae

rs0162 6 2109.43 956394 977.954 0.00000 -2.15676 Lachnospiraceae

#rs0111 6 5849.41 8.12613e+06 2850.64 45.64334 -2.03588 Lachnospiraceae

rs0199 6 1549.24 605556 778.175 0.00000 -1.9906 Desulfovibrionaceae

rs0034 6 22703 1.39693e+08 11819.2 0.00000 -1.92084 unknown

#rs0054 6 13289.5 5.32369e+07 7296.36 0.00000 -1.82137 Lachnospiraceae

rs0141 6 3013.72 2.71102e+06 1646.52 30.42890 -1.81177 Fibrobacteraceae

rs0012 6 51311.4 1.0194e+09 31928.1 0.00000 -1.60709 Veillonellaceae

rs0139 6 3703.53 5.59732e+06 2365.86 0.00000 -1.56534 Selenomonadaceae

rs0077 6 554.63 97408.7 312.104 76.07224 -1.53284 Acidaminococcaceae

rs0192 6 2866.08 3.61149e+06 1900.39 0.00000 -1.50807 Selenomonadaceae

rs0097 6 8908.24 3.51807e+07 5931.33 0.00000 -1.50187 Lachnospiraceae

rs0027 6 24973.6 3.13359e+08 17701.9 0.00000 -1.41078 Prevotellaceae

rs0190 6 3446.68 6.14914e+06 2479.75 0.00000 -1.38988 Lachnospiraceae

rs0063 6 3626.32 6.83936e+06 2615.22 15.21445 -1.38075 Lachnospiraceae

rs0009 6 54167.9 1.56476e+09 39557 76.07224 -1.36744 Prevotellaceae

rs0103 6 6462.68 2.31933e+07 4815.94 0.00000 -1.34191 Prevotellaceae

#rs0215 6 2961.1 5.10221e+06 2258.81 0.00000 -1.31086 Selenomonadaceae

rs0075 6 7507.25 3.90521e+07 6249.17 0.00000 -1.2013 Prevotellaceae

rs0008 6 42135 1.28428e+09 35836.9 15.21445 -1.17532 Prevotellaceae

rs0179 6 3853.33 1.07726e+07 3282.17 0.00000 -1.17399 Lachnospiraceae

rs0104 6 7363.02 3.99354e+07 6319.45 0.00000 -1.16512 Clostridia_UCG-014

rs0297 6 39.2974 1262.04 35.5252 0.00000 -1.10308 Prevotellaceae

rs0225 6 3063.94 7.88804e+06 2808.57 0.00000 -1.09089 Selenomonadaceae

#rs0090 6 8871.26 6.59626e+07 8121.74 30.42890 -1.08853 Selenomonadaceae

rs0014 6 50122.4 2.19255e+09 46824.6 30.42890 -1.06978 Lachnospiraceae

rs0013 6 53526.4 2.70295e+09 51989.9 0.00000 -1.02955 Prevotellaceae

rs0017 6 60731.6 3.59344e+09 59945.3 0.00000 -1.01311 Bifidobacteriaceae

rs0031 6 23058.9 5.30095e+08 23023.8 0.00000 -1.00152 Prevotellaceae

**CepravinLC/cn2319**, 20 most changed OTU)

OTU count mean variance std_dev cn2319 z-score Family

rs0116 7 1239.21 50372 224.437 722.02166 -5.51893 Oscillospiraceae

rs0243 7 1147.57 66749.2 258.359 604.14057 -4.44004 Prevotellaceae

rs0265 7 828.681 37508 193.67 279.96758 -4.27662 F082

rs0079 7 1447.22 167299 409.022 5348.85434 -3.53739 Selenomonadaceae

rs0135 7 1993.91 351472 592.851 559.93517 -3.36268 Fibrobacteraceae

rs0115 7 1572.67 226597 476.022 1178.81087 -3.30308 Lachnospiraceae

rs0030 7 7348.68 5.53559e+06 2352.78 4582.62727 -3.12326 Acidaminococcaceae

rs0239 7 959.462 95457 308.961 221.02704 -3.10444 Ruminococcaceae

rs0177 7 1089.77 125511 354.276 648.34598 -3.07518 F082

rs0019 7 11726.7 1.50013e+07 3873.15 49126.94320 -3.0276 Selenomonadaceae

rs0241 7 239.942 6785.37 82.3734 147.35136 -2.90933 Prevotellaceae

rs0081 7 942.761 110795 332.859 1974.50821 -2.83146 Rikenellaceae

rs0005 7 33543.9 1.43219e+08 11967.4 24150.88779 -2.80291 Acidaminococcaceae

rs0178 7 1126.04 163245 404.036 1119.87033 -2.7863 Hungateiclostridiaceae

rs0134 7 1732.82 405918 637.117 1399.83791 -2.71936 Lachnospiraceae

rs0010 7 17981.4 4.42686e+07 6653.47 17475.87121 -2.70252 Prevotellaceae

rs0144 7 2032.7 579819 761.459 5378.32462 -2.66913 Selenomonadaceae

rs0124 7 1805.73 470190 685.704 1296.69196 -2.63301 Muribaculaceae

rs0107 7 1642.66 395599 628.967 1370.36764 -2.61127 Christensenellaceae

rs0236 7 663.365 66371.4 257.627 442.05408 -2.57391 Prevotellaceae

# OTU with high negative MeY correlations
